# Carotid axillary bypass in a patient with blocked subclavian stents: a case report

**DOI:** 10.1186/1752-1947-5-237

**Published:** 2011-06-27

**Authors:** Tarig I Barakat, Louise Kenny, Hazim Khout, Grace Timmons, Vish Bhattacharya

**Affiliations:** 1 Department of General Surgery, Queen Elizabeth Hospital, Sheriff Hill, Gateshead, Tyne & Wear, NE9 6SX, UK

## Abstract

**Introduction:**

Surgical treatment of symptomatic occlusive lesions of the proximal subclavian artery is infrequently necessary. Carotid subclavian bypass has gained popularity and is now considered standard treatment when stenting is not possible. Exposure of the subclavian artery and bypass grafting onto it is difficult, as the vessel is delicate, thin-walled and located deep in the supraclavicular fossa. The thoracic duct and brachial plexus are in close proximity to the left subclavian artery and are therefore susceptible to damage. Distal grafting to the axillary artery instead of the subclavian artery has the potential of avoiding some of these risks. Infraclavicular exposure of the axillary artery is more straightforward. The vessel wall is thicker and is easier to handle. In this case report, we describe a patient with a left proximal subclavian occlusion which was stented twice and blocked on both occasions. The patient underwent a carotid axillary bypass, as grafting onto the subclavian artery was impossible because of the two occluded metal stents.

**Case presentation:**

A 56-year-old Caucasian woman, a heavy smoker, presented acutely with left arm numbness and pain and blood pressure discrepancies in both arms. A diagnosis of subclavian stenosis was confirmed on the basis of a computed tomographic scan and a magnetic resonance angiogram. The patient had undergone subclavian artery stenting twice, and unfortunately the stents blocked on both occasions. The patient underwent carotid axillary bypass surgery. She had an uneventful recovery and was able to return to a full, normal life.

**Conclusion:**

Carotid axillary bypass appears to be a good alternative to carotid subclavian bypass in the treatment of symptomatic proximal stenosis or occlusion of the subclavian artery.

## Introduction

Although proximal subclavian artery disease is often asymptomatic, once ischemic or embolic complications occur, surgery may be necessary. Transluminal therapy of lesions of subclavian, innominate and common carotid arteries by balloon angioplasty, with or without stenting, is an increasingly performed procedure, especially in cases of stenosis.

Although preliminary data for focal lesions are encouraging, careful reporting of long-term results will be the only way to determine whether these non-surgical endoluminal procedures are sufficiently effective to be offered as reasonable alternatives to the better-proven surgical reconstructions.

The use of extrathoracic reconstruction for patients with symptomatic proximal subclavian artery disease is well-established. The carotid subclavian bypass is the commonest surgical procedure in cases in which stenting is not possible.

This procedure was first described by Diethrich *et al*. in 1967 [1], and excellent long-term results have been described in several case series [2-8]

Exposure of the subclavian artery carries with it the potential risk of damage to major lymphatic vessels and nerves. Exposure of the axillary artery using the infraclavicular approach is technically easier. The artery is easier to handle, and the wall of the vessel is thicker. However, there is a small risk of brachial plexus damage.

Criado [9] performed 26 carotid-axillary surgical procedures in 10 years, and he reported 96% graft patency rate over four years. He used prosthetic Dacron and polytetrafluoroethylene (PTFE) ringed grafts tunneled under the clavicle. No shunting is needed unless the patient has a significant internal carotid lesion. The chance of distal embolization is minimal.

## Case report

We describe the case of a 56-year-old Caucasian woman who presented acutely with left arm numbness and pain lasting for nearly eight hours. She had had similar episodes of numbness a few days previously, but these had lasted for only five to 10 minutes each time.

She smoked 20 cigarettes/day. Her heart rate was regular, although her blood pressure was lower in the left arm than in the right arm (103/80 mmHg vs. 170/80 mmHg, respectively). Her left arm looked pink but cool, with no palpable brachial, radial or ulnar pulse. There was decreased sensation over the forearm, though there was no motor deficit.

Her chest, cardiovascular and abdominal examinations showed no other abnormalities. She underwent urgent computed tomography, which showed an acutely thrombosed left subclavian artery.

**Figure 1:**
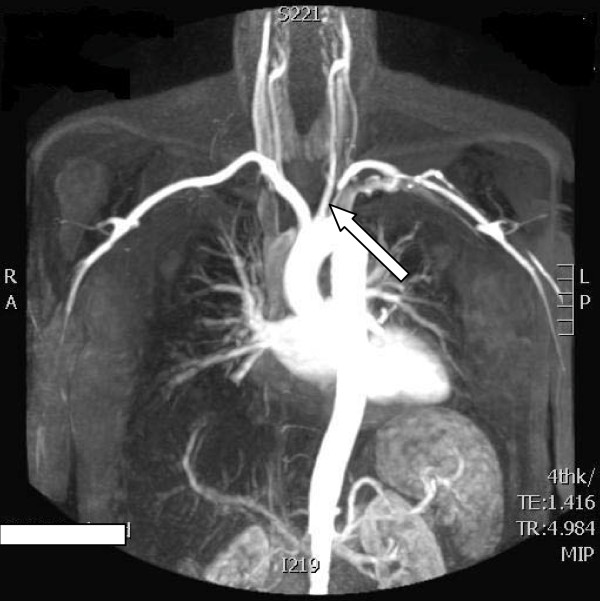
**Magnetic resonance angiogram shows stenosis of the proximal left subclavian artery**. Arrow shows area of proximal subclavian artery stenosis.

**Figure 2:**
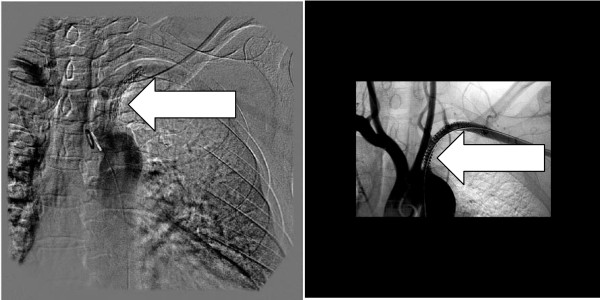
**(A)The first stent was placed successfully**. **(B) **The second Nitinol stent was placed within the first stent after 3 months.

She was put on an intravenous heparin infusion and magnetic resonance angiography was arranged (Figure 1). Initial angiograms obtained through the femoral artery in the groin showed a tight stenosis which was right at the origin of the subclavian artery. As a result, a guidewire could not be passed through the groin puncture despite several attempts. The brachial route was therefore chosen. A guidewire was passed using a left brachial artery approach through the narrowing. A 5 mm × 4 cm stainless steel stent Genesis (Cordis Endovascular, Warren, NJ, USA) was subsequently deployed and, when ballooned, although it clearly had eliminated the atherosclerotic lesion, the diameter was less than the diameter of the native normal vessel. To improve conformity, the stent was ballooned to 6 mm, which improved the conformity. A good, brisk flow through the stent was confirmed, and the procedure was subsequently completed (Figure 2A).

Unfortunately, the patient continued to smoke heavily and was soon re-admitted with recurrent symptoms. The duplex repeat angiogram confirmed an occlusion of the left subclavian stent. The occlusion was successfully traversed, and a 6 cm-long, 7 mm S.M.A.R.T. Nitinol Stent System (Cordis Corporation, Miami Lakes, FL, USA) was deployed through the original stent with a good result (Figure 2B).

Unfortunately, the stent blocked again for the second time, and a decision made to carry out a bypass rather than perform repeat radiological re-intervention. She therefore underwent a carotid to axillary bypass.

**Figure 3:**
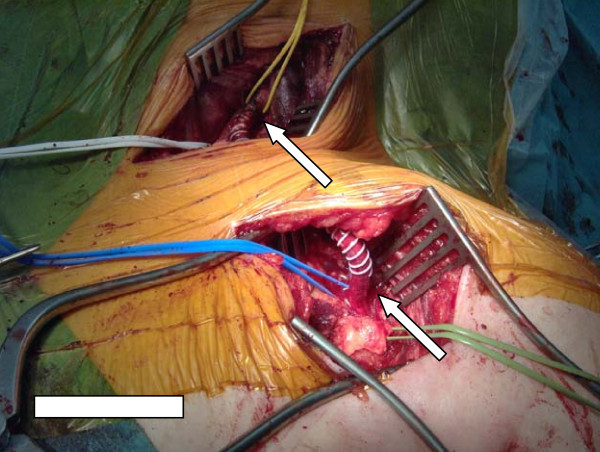
**Carotid axillary bypass using PTFE graft**. Arrows show anastomosis sites.

Intra-operatively, the left common carotid artery was approached through a longitudinal incision along the medial aspect of the left sternocleidomastoid (SCM). The artery was exposed and controlled. The left axillary artery was approached through an infraclavicular incision parallel to the clavicle. The artery was exposed and controlled. A total of 3000IU of heparin were given intravenously. A 6 mm ringed PTFE graft was tunneled under the SCM and over the clavicle and anastomosed using a 5-0 Prolene suture (Figure 3). Good pulses were established after the procedure. The patient had an uneventful recovery, and her claudication symptoms settled completely. At her one-year follow-up examination, the graft was still patent and she was asymptomatic.

## Discussion

Extrathoracic revascularization is an effective and safe way to treat branch occlusions of the aortic arch. The carotid-subclavian bypass in particular has received much-deserved attention since the report by Diethrich *et al*. [1]. Its excellent long-term results have been duplicated by several groups around the world. That notwithstanding, the potential hazards and difficulties of subclavian artery exposure should be further emphasized. The proximity of major lymphatic structures may pose technical difficulties and increase the risk of complications. In this particular case, the graft was tunneled over the clavicle in favor of the retroclavicular approach, as it was anticipated that, because of the repeated stenting trials in this patient, there could be fibrosis and local inflammation which would make dissection difficult with increased risk of injury to the subclavian vein. Encouraged by early reports [8,10], we postulate that use of the axillary artery as the distal anastomotic site would simplify the operation and avoid the risk of lymphatic injury altogether.

Carotid axillary bypass is a very good alternative to carotid subclavian bypass. In this case specifically, the carotid axillary bypass was favored because of the presence of stents in the subclavian artery, which would have made grafting very difficult.

The risk of operation-related stroke is very minimal. Shunting is unnecessary unless a critical internal carotid lesion co-exists on the same side. Concomitant carotid endarterectomy at the donor graft site may be performed in patients with severe atheromatous plaques. With regard to radiology, attempted recanalization for subclavian arteries is better approached from a brachial puncture than from a groin puncture.

## Conclusion

Carotid axillary bypass is a very good alternative to carotid subclavian bypass. It is safe and technically easier, and it provides equally good short- and long-term results.

## Consent

Written informed consent was obtained from the patient for publication of this case report and any accompanying images. A copy of the written consent is available for review by the editor in chief of this journal.

## Competing interests

The authors declare that they have no competing interests.

## Authors' contributions

TB was involved in the major parts of writing the paper and performing the literature search, as well as being involved in performing the surgery and in the patient's pre-operative and post-operative care. LK and HK contributed to writing the manuscript and to the literature search. GT was involved in the radiological procedures. VB was the responsible vascular surgeon and team leader who set the management plan. All authors read the manuscript and agreed to its contents.
